# Peroxisome Proliferator-Activated Receptors (PPARs): A Themed Issue in Honor of Prof. Walter Wahli

**DOI:** 10.3390/biom15091276

**Published:** 2025-09-03

**Authors:** Hervé Guillou, Manuel Vázquez-Carrera

**Affiliations:** 1 INRAE Toulouse, Toxalim Unit, 180 Chemin de Tournefeuille, BP93173, 31027 Toulouse, France; 2 Department of Pharmacology, Toxicology and Therapeutic Chemistry, Faculty of Pharmacy and Food Sciences, 08028 Barcelona, Spain; 3 Institute of Biomedicine of the University of Barcelona (IBUB), University of Barcelona, 08028 Barcelona, Spain; 4 Spanish Biomedical Research Center in Diabetes and Associated Metabolic Diseases (CIBERDEM)-Instituto de Salud Carlos III, 28029 Madrid, Spain; 5 Pediatric Research Institute-Hospital Sant Joan de Déu, 08950 Esplugues de Llobregat, Spain

It is with great pleasure that we introduce this Special Issue dedicated to Peroxisome Proliferator-Activated Receptors (PPARs), honoring the pioneering contributions of **Professor Walter Wahli**. Over the course of more than four decades, Prof. Wahli has significantly contributed to our understanding of nuclear receptor biology, metabolic regulation, and the intersection of lipid signaling and inflammation. His work spans from foundational mechanistic insights to concepts that underpin today’s translational efforts in metabolic, inflammatory, and cardiovascular diseases. Beyond his discoveries, Prof. Wahli’s enthusiasm, generosity, and commitment to collaborative science have inspired generations of researchers worldwide.

## 1. From Vitellogenin to Nuclear Receptor Mechanisms

Prof. Wahli’s scientific journey began with elegant studies in Xenopus laevis that established the liver vitellogenin genes that encode yolk proteins as hormone-responsive target models. As a Ph.D. researcher in Rudolf Weber’s laboratory (University of Berne, Switzerland), he was the first to isolate the vitellogenin mRNA [1] using sucrose gradients, and, in collaboration with a postdoc in the laboratory, Gerhart Ryffel, he titrated vitellogenin mRNA accumulation in the liver of estrogen-treated male *Xenopus laevis*, using C_o_t curves [2]. These two papers were the core of his PhD thesis at the University of Berne, which was completed in only two years (1975–1977) and for which he received a “*Summa cum laude*”. After his Ph.D., he pursued his quest for the molecular mechanism of estrogen action during his postdoctoral stage in Igor Dawid’s laboratory (Department of Embryology of the Carnegie Institution, Baltimore, MD, USA) and then at the National Institutes of Health, Bethesda, where he cloned vitellogenin mRNA [3] and built the first Xenopus genomic library. From this library, he isolated the vitellogenin genes and found, a big surprise at the time, that these genes were organized into not less than 33 exons and 32 introns [4,5,6]. This finding was highlighted in the front-page picture of the Cell issue in which his 1980 paper [5] was published.

Upon returning to Switzerland as a Full Professor and Director of the Institute of Animal Biology at the University of Lausanne, Walter Wahli’s group defined the canonical estrogen response element (ERE) from a comparative study of the promoter of the estrogen-responsive vitellogenin and apo-VLDLI genes, characterized its sequence requirements and cooperative receptor binding, and demonstrated hormone-dependent transcription using a technically demanding homologous in vitro system [7,8,9,10,11,12,13,14]. Collectively, these landmark contributions showed that the estrogen receptor resides in the nucleus in an inactive state and, upon ligand binding, drives transcription by engaging EREs in target promoters, principles that became pillars of nuclear receptor biology.

## 2. Founding and Expanding the PPAR Field

In the late 1980s, Prof. Wahli and one of his postdoctoral fellows, Greg Krey, in collaboration with Christine Dreyer (Tuebingen, Germany), applied the estrogen receptor DNA-binding domain as a probe to uncover novel receptors, leading to the discovery and characterization of the PPAR subfamily (PPARα, PPARβ/δ, and PPARγ) [15], for which a first member was discovered independently by Stephen Green [16]. This work, together with seminal ligand studies, established that fatty acids and eicosanoids directly modulate gene expression through PPARs, marking a paradigm shift that linked nutrient lipids to transcriptional control [17,18]. This significant finding opened a new field of investigation of the highest biomedical significance. A postdoc in Walter Wahli’s research group, Pallavi Devchand, was the first to demonstrate that the fibrate hypolipidemic drugs, already on the market for some time, can directly bind to PPARα, which unveiled the mode of action of these drugs in lowering circulating lipids and uncovered the involvement of PPARα in the control of inflammation [19]. These findings prompted them to investigate the metabolic response mammals have evolved to survive long periods of energy deprivation. Indeed, a postdoctoral fellow in Wahli’s group, Sander Kersten, demonstrated that PPARα plays a pivotal role in regulating energy stores during fasting. By modulating gene expression, PPARα stimulates hepatic fatty acid oxidation, providing energy-rich substrates that can be metabolized by peripheral organs [20].

## 3. Physiological Breadth: From Tissue Repair to Cancer and Cardiometa-bolism

Prof. Wahli’s research continuously broadened the physiological canvas of PPARs. In the skin, a novel discovery by his group, initiated by postdoctoral fellow Liliane Michalik, revealed impaired wound healing in PPARα- and PPARβ/δ-deficient mice [21]. Building on this, and in collaboration with Andrew Tan, they identified a homeostatic control of keratinocyte proliferation and differentiation mediated through a PPAR-driven response in dermal fibroblasts via interleukin 1 (IL-1) signaling [22]. These findings have also opened an avenue for investigating the possible implications of PPARβ/δ in controlling tumor development [23].

Expanding from skin to metabolic tissues, genetic tools developed in collaboration with Pierre Chambon and Daniel Metzger, within the framework of a Human Frontier Science Program project directed by Walter Wahli, allowed them to demonstrate that PPARγ was required in differentiated, mature white and brown adipocytes for their survival [24]. In parallel, in the vasculature, collaboration with Philippe Boucher uncovered that PPARγ protects against calcification by antagonizing Wnt Family Member 5A (WNT5A) signaling, a finding with direct clinical relevance for atherosclerosis and valvular disease [25]. Extending into skeletal muscle physiology, Profs. Wahli and Chambon’s teams demonstrated that PPARβ/δ plays a crucial role in maintaining oxidative muscle fibers, with its deficiency leading to fiber-type switching, obesity, and diabetes [26]. Likewise, in the heart, collaboration with Manuel Vázquez-Carrera revealed that PPARβ/δ activation counteracts phenylephrine-induced cardiomyocyte hypertrophy in neonatal rat ventricular cardiomyocytes [27]. Finally, in the endocrine pancreas, Wahli’s group demonstrated that epithelial PPARβ/δ constrains β-cell mass and exocytosis, unveiling a regulatory checkpoint in insulin secretion [28]. In addition, sex-dependent repression by sumoylated PPARα in the female liver offered a mechanistic framework for estrogen-linked cholestatic disease [29]. Together, these studies demonstrate how PPARs orchestrate cellular and systemic physiology across diverse tissues and organs.

## 4. Developmental and Nutritional Axes

A hallmark of Prof. Wahli’s contributions is the integration of developmental, nutritional, and epigenetic dimensions. He uncovered a glucocorticoid receptor–PPARα axis in fetal liver that equips neonates for milk lipid catabolism, including epigenetic gating of fibroblast growth factor 21 (FGF21) responsiveness [30]. In collaboration with Hervé Guillou and Catherine Postic, they revealed a specific PPARα–carbohydrate-responsive element-binding protein (ChREBP) cross-talk required for glucose-mediated FGF21 induction and endocrine control of sugar intake [31] and established hepatocyte PPARα as a protector against metabolic dysfunction-associated steatotic liver disease (MASLD) and hypercholesterolemia during aging [32]. In the intestine, collaboration with Philippe Sansonetti demonstrated that a high-fat diet disrupts PPARγ-dependent epithelial-microbial homeostasis [33]. In contrast, caloric restriction polarizes mucosal programs with PPARα and interferon-stimulated gene factor 3 (ISGF3) as potential candidate regulators [34]. Extending these insights to systems physiology, collaborative work with Sven Pettersson demonstrated that the gut microbiota can influence skeletal muscle mass and function [35].

## 5. PPARs as Nutrient-Sensing Transcriptional Hubs Coordinating Inter-Organ Communication and Stress Cytokines

In recent years, Prof. Wahli’s work, mainly collaborations with Hervé Guillou and Manuel Vázquez-Carrera, has focused on PPAR control of systemic metabolism and the regulation of stress cytokines. These collaborations have built on the understanding that intact PPARα activity in hepatocytes is required for the crosstalk between adipose tissues and the liver during fat mobilization [36] and why metabolic control by PPARα in hepatocytes plays a crucial role in host defense against infection [37]. In addition, these collaborations have contributed to elucidating that growth differentiation factor 15 (GDF15) mediates the metabolic effects of PPARβ/δ by activating AMPK [38] and that PPARβ/δ prevents inflammation and fibrosis during diabetic cardiomyopathy [39]. All these studies confirm that PPARs serve as nutrient-sensing transcriptional hubs that coordinate inter-organ communication to maintain metabolic and inflammatory homeostasis. Prof. Wahli’s mentorship and scientific imagination remain central drivers of these achievements.

## 6. About This Special Issue

The articles assembled in this Special Issue mirror the diversity and translational promise of the PPAR field. Contributions span basic mechanisms of receptor–ligand interaction and endogenous activation; structural and epigenetic regulation; tissue- and species-specific roles (e.g., lung, heart, skin, and ray-finned fish); natural ligand and modulator pharmacology; and broad therapeutic or translational applications in areas such as cardiovascular function, metabolic disorders, skin pathology, and clinical perspectives on PPAR biology. In curating these works, our aim is twofold: to celebrate Prof. Wahli’s foundational role and to chart the questions that will define the next chapter of the PPAR research journey (precision targeting of PPAR networks, context-aware modulation, and integrative systems approaches bridging molecules to medicine).

## 7. Concluding Remarks

Prof. Walter Wahli’s scientific legacy is distinguished by originality, rigor, and a gift for connecting dots across biology (from amphibian vitellogenesis to mammalian lactation, and from nuclear receptor mechanics to organismal homeostasis). Equally, it is a story of collaboration. Many of the advances highlighted here were made possible by enduring partnerships (including Béatrice Desvergne, Liliane Michalik, Pierre Chambon, Nguan Soon Tan, Hervé Guillou, and Manuel Vázquez-Carrera) that exemplify the best of our community. On behalf of all contributors, we express our deep gratitude to Prof. Wahli for his leadership, inspiration, and friendship. May this Special Issue honor his achievements and energize future discoveries.
